# *o*-Benzoquinone dioxime

**DOI:** 10.1107/S1600536810039619

**Published:** 2010-10-09

**Authors:** Giuliana Gervasio, Domenica Marabello, Federica Bertolotti

**Affiliations:** a Dipartimento di Chimica I.F.M.,University of Turin, Via P. Giuria 7, 10125, Torino, Italy

## Abstract

The title compound, C_6_H_6_N_2_O_2_, was obtained as a product of an *in vitro* study of the metabolism of benzofuroxan. The  molecule exhibits a *amphi* configuration of the oxime groups C=N—OH. One oxime group is involved in the formation of a strong intra­molecular O—H⋯N hydrogen bond, while another links mol­ecules into zigzag chains along the *c* axis *via* inter­molecular O—H⋯N hydrogen bonds.

## Related literature

For details of the synthesis, see: Grosa *et al.* (2004 ▶). For a related structure, see: Mégnamisi-Bélombé & Endres (1985 ▶). 
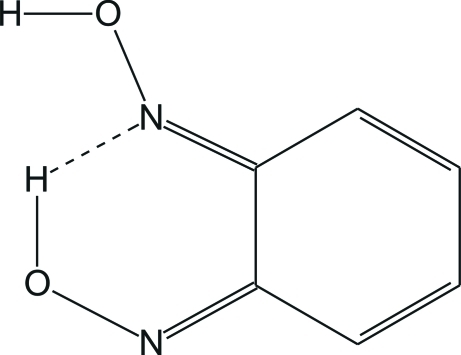


## Experimental

### 

#### Crystal data

C_6_H_6_N_2_O_2_
*M* *_r_* = 138.13
Orthorhombic, 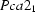

*a* = 15.009 (5) Å
*b* = 3.8181 (13) Å
*c* = 10.694 (3) Å
*V* = 612.8 (4) Å^3^
*Z* = 4
Mo *K*α radiation
μ = 0.12 mm^−1^
*T* = 293 K
0.24 × 0.12 × 0.04 mm

#### Data collection

Siemens–Bruker APEX diffractometer
Absorption correction: multi-scan (Blessing, 1995 ▶) *T*_min_ = 0.856, *T*_max_ = 1.000
2330 measured reflections
468 independent reflections
418 reflections with *I* > 2σ(*I*)
*R*_int_ = 0.055
θ_max_ = 23.3°
11 standard reflections every 60 min  intensity decay: none

#### Refinement

*R*[*F* ^2^ > 2σ(*F* ^2^)] = 0.034
*wR*(*F* ^2^) = 0.082
*S* = 1.01
468 reflections
99 parameters
1 restraint
H atoms treated by a mixture of independent and constrained refinement
Δρ_max_ = 0.19 e Å^−3^
Δρ_min_ = −0.13 e Å^−3^

### 

Data collection: *SMART* (Bruker, 2007 ▶); cell refinement: *SAINT* (Bruker, 2007 ▶); data reduction: *SAINT*; program(s) used to solve structure: *SHELXS97* (Sheldrick, 2008 ▶); program(s) used to refine structure: *SHELXL97* (Sheldrick, 2008 ▶); molecular graphics: *SHELXTL* (Sheldrick, 2008 ▶); software used to prepare material for publication: *SHELXL97*.

## Supplementary Material

Crystal structure: contains datablocks I, global. DOI: 10.1107/S1600536810039619/cv2766sup1.cif

Structure factors: contains datablocks I. DOI: 10.1107/S1600536810039619/cv2766Isup2.hkl

Additional supplementary materials:  crystallographic information; 3D view; checkCIF report

## Figures and Tables

**Table 1:** Hydrogen-bond geometry (Å, °)

*D*—H⋯*A*	*D*—H	H⋯*A*	*D*⋯*A*	*D*—H⋯*A*
O1—H1⋯N2^i^	0.85 (7)	1.92 (7)	2.745 (4)	162 (6)
O2—H2⋯N1	1.06 (8)	1.57 (8)	2.532 (4)	147 (6)

Symmetry code: (i) 
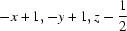
.

**Fig. 1.**
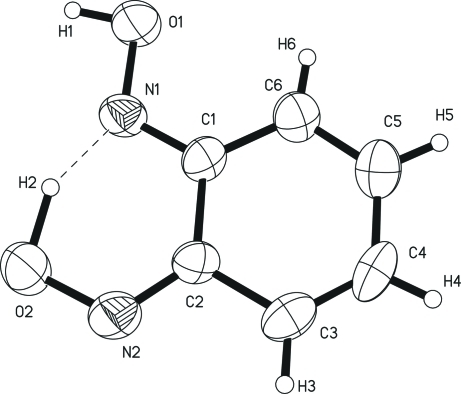
The molecular structure of trhe title compound showing the atomic numbering and 50% of probability displacements ellipsoids.

## supplementary crystallographic information

### Comment

The title compound, o-benzoquinone dioxime, has been obtained according to Grosa *et al.* (2004). In the C1—C6 ring the C3-C4 and C5-C6 bond distances correspond to formal double bonds (1.336 (5) Å av.). Also the C1-N1 and C2-N2 distances agree with a double bond character (1.304 (5) Å av.). Noteworthy is the presence of a strong intramolecular hydrogen bond O2-H2···N2 that probably stabilize the syn form of the dioxime. A further intermolecular hydrogen bond O1-H1..N2 forms chains of molecules. O-benzoquinone dioxime is known as an excellent ligand which forms bis-chelated transition metal complexes especially with the dipositive metal ions of the Ni triad (cf. Mégnamisi-Bélombé & Endres, 1985).

### Experimental

The *o*-benzoquinone dioxime has been otained according to Grosa *et al.* (2004)

### Refinement

A very small and poorly diffracting crystal has been used; it was not possible to obtain a better crystal because it is a product of a metabolism. C-bound H atoms were placed in geometrically idealized positions (C—H = 0.93 Å), and refined as riding, with *U*_iso_(H) = 1.2*U*_eq_(C). Two O-bound H atoms were located on a difference map and refined isotropically. A restraint has been imposed on the planarity of the hexagonal ring. In the absence of any significant anomalous scatterers in the molecule, 368 Friedel pairs were merged before the final refinement.

### Crystal data

**Table:**

C_6_H_6_N_2_O_2_	*D*_x_ = 1.497 Mg m^−^^3^
*M**_r_* = 138.13	Mo *K*α radiation, λ = 0.71073 Å
Orthorhombic, *P**c**a*2_1_	Cell parameters from 500 reflections
*a* = 15.009 (5) Å	θ = 2.7–23.3°
*b* = 3.8181 (13) Å	µ = 0.12 mm^−^^1^
*c* = 10.694 (3) Å	*T* = 293 K
*V* = 612.8 (4) Å^3^	Prism, orange
*Z* = 4	0.24 × 0.12 × 0.04 mm
*F*(000) = 288	

### Data collection

**Table:**

Siemens–Bruker APEX diffractometer	418 reflections with *I* > 2σ(*I*)
Radiation source: fine-focus sealed tube	*R*_int_ = 0.055
graphite	θ_max_ = 23.3°, θ_min_ = 2.7°
φ scans	*h* = −16→16
Absorption correction: multi-scan (Blessing, 1995)	*k* = −4→3
*T*_min_ = 0.856, *T*_max_ = 1.000	*l* = −11→11
2330 measured reflections	11 standard reflections every 60 min
468 independent reflections	intensity decay: none

### Refinement

**Table:**

Refinement on *F*^2^	Primary atom site location: structure-invariant direct methods
Least-squares matrix: full	Secondary atom site location: difference Fourier map
*R*[*F*^2^ > 2σ(*F*^2^)] = 0.034	Hydrogen site location: inferred from neighbouring sites
*wR*(*F*^2^) = 0.082	H atoms treated by a mixture of independent and constrained refinement
*S* = 1.01	*w* = 1/[σ^2^(*F*_o_^2^) + (0.0605*P*)^2^] where *P* = (*F*_o_^2^ + 2*F*_c_^2^)/3
468 reflections	(Δ/σ)_max_ < 0.001
99 parameters	Δρ_max_ = 0.19 e Å^−^^3^
1 restraint	Δρ_min_ = −0.13 e Å^−^^3^

### Special details

**Table:**

Geometry. All e.s.d.'s (except the e.s.d. in the dihedral angle between two l.s. planes) are estimated using the full covariance matrix. The cell e.s.d.'s are taken into account individually in the estimation of e.s.d.'s in distances, angles and torsion angles; correlations between e.s.d.'s in cell parameters are only used when they are defined by crystal symmetry. An approximate (isotropic) treatment of cell e.s.d.'s is used for estimating e.s.d.'s involving l.s. planes.
Refinement. Refinement of *F*^2^ against ALL reflections. The weighted *R*-factor *wR* and goodness of fit *S* are based on *F*^2^, conventional *R*-factors *R* are based on *F*, with *F* set to zero for negative *F*^2^. The threshold expression of *F*^2^ > σ(*F*^2^) is used only for calculating *R*-factors(gt) *etc*. and is not relevant to the choice of reflections for refinement. *R*-factors based on *F*^2^ are statistically about twice as large as those based on *F*, and *R*- factors based on ALL data will be even larger.

### Fractional atomic coordinates and isotropic or equivalent isotropic displacement parameters (Å^2^)

**Table:**

	*x*	*y*	*z*	*U*_iso_*/*U*_eq_	
C1	0.6109 (3)	0.3317 (8)	0.4796 (3)	0.0377 (8)	
C2	0.5481 (2)	0.2457 (9)	0.5795 (3)	0.0377 (8)	
C3	0.5843 (3)	0.0751 (9)	0.6896 (3)	0.0479 (10)	
H3A	0.5461	0.0119	0.7543	0.057*	
C4	0.6706 (3)	0.0072 (9)	0.6998 (3)	0.0520 (12)	
H4A	0.6919	−0.1043	0.7711	0.062*	
C5	0.7312 (3)	0.1027 (10)	0.6031 (3)	0.0532 (10)	
H5A	0.7916	0.0552	0.6128	0.064*	
C6	0.7028 (3)	0.2599 (9)	0.4981 (3)	0.0461 (9)	
H6A	0.7437	0.3225	0.4368	0.055*	
N1	0.5761 (2)	0.4735 (7)	0.3794 (3)	0.0426 (8)	
N2	0.4629 (2)	0.3068 (8)	0.5828 (3)	0.0489 (8)	
O1	0.6388 (2)	0.5573 (8)	0.2905 (2)	0.0561 (8)	
H1	0.603 (4)	0.638 (17)	0.236 (6)	0.11 (2)*	
O2	0.4235 (2)	0.4598 (7)	0.4801 (2)	0.0584 (9)	
H2	0.477 (5)	0.541 (17)	0.422 (6)	0.13 (2)*	

### Atomic displacement parameters (Å^2^)

**Table:**

	*U*^11^	*U*^22^	*U*^33^	*U*^12^	*U*^13^	*U*^23^
C1	0.045 (2)	0.0412 (18)	0.0267 (15)	−0.0013 (15)	−0.0039 (15)	−0.0047 (13)
C2	0.043 (2)	0.0426 (19)	0.0278 (15)	−0.0044 (16)	0.0022 (16)	−0.0053 (14)
C3	0.067 (3)	0.046 (2)	0.0306 (17)	−0.0030 (17)	0.0005 (18)	−0.0007 (18)
C4	0.072 (3)	0.050 (2)	0.035 (2)	0.004 (2)	−0.018 (2)	0.0031 (14)
C5	0.054 (3)	0.055 (2)	0.050 (2)	0.0067 (19)	−0.012 (2)	−0.0059 (17)
C6	0.047 (2)	0.053 (2)	0.0382 (18)	0.0039 (18)	−0.0018 (17)	−0.0051 (17)
N1	0.041 (2)	0.0576 (19)	0.0294 (14)	−0.0035 (13)	0.0044 (16)	−0.0011 (12)
N2	0.051 (2)	0.0646 (18)	0.0306 (14)	0.0003 (17)	0.0034 (15)	−0.0015 (16)
O1	0.0470 (18)	0.091 (2)	0.0303 (12)	−0.0017 (14)	0.0032 (14)	0.0104 (13)
O2	0.046 (2)	0.090 (2)	0.0393 (14)	0.0042 (14)	−0.0021 (14)	0.0018 (13)

### Geometric parameters (Å, °)

**Table:**

C1—N1	1.309 (5)	C4—H4A	0.9300
C1—C6	1.420 (5)	C5—C6	1.342 (5)
C1—C2	1.462 (5)	C5—H5A	0.9300
C2—N2	1.299 (4)	C6—H6A	0.9300
C2—C3	1.450 (5)	N1—O1	1.375 (4)
C3—C4	1.326 (6)	N2—O2	1.377 (4)
C3—H3A	0.9300	O1—H1	0.85 (7)
C4—C5	1.425 (6)	O2—H2	1.06 (8)
			
N1—C1—C6	125.5 (3)	C5—C4—H4A	119.5
N1—C1—C2	115.8 (3)	C6—C5—C4	121.2 (4)
C6—C1—C2	118.7 (3)	C6—C5—H5A	119.4
N2—C2—C3	115.3 (3)	C4—C5—H5A	119.4
N2—C2—C1	127.8 (3)	C5—C6—C1	120.8 (4)
C3—C2—C1	116.9 (3)	C5—C6—H6A	119.6
C4—C3—C2	121.4 (4)	C1—C6—H6A	119.6
C4—C3—H3A	119.3	C1—N1—O1	112.9 (3)
C2—C3—H3A	119.3	C2—N2—O2	118.5 (3)
C3—C4—C5	120.9 (3)	N1—O1—H1	97 (4)
C3—C4—H4A	119.5	N2—O2—H2	105 (4)

### Hydrogen-bond geometry (Å, °)

**Table:**

*D*—H···*A*	*D*—H	H···*A*	*D*···*A*	*D*—H···*A*
O1—H1···N2^i^	0.85 (7)	1.92 (7)	2.745 (4)	162 (6)
O2—H2···N1	1.06 (8)	1.57 (8)	2.532 (4)	147 (6)

Symmetry codes: (i) −*x*+1, −*y*+1, *z*−1/2.
